# Assessment and correction of macroscopic field variations in 2D spoiled gradient‐echo sequences

**DOI:** 10.1002/mrm.28139

**Published:** 2019-12-23

**Authors:** Martin Soellradl, Andreas Lesch, Johannes Strasser, Lukas Pirpamer, Rudolf Stollberger, Stefan Ropele, Christian Langkammer

**Affiliations:** ^1^ Department of Neurology Medical University of Graz Graz Austria; ^2^ Institute of Medical Engineering Graz University of Technology Graz Austria

**Keywords:** field inhomogeneities, myelin water fraction, $R_{2}^{*}$, relaxometry, slice profile, $T_{2}^{*}$

## Abstract

**Purpose:**

To model and correct the dephasing effects in the gradient‐echo signal for arbitrary RF excitation pulses with large flip angles in the presence of macroscopic field variations.

**Methods:**

The dephasing of the spoiled 2D gradient‐echo signal was modeled using a numerical solution of the Bloch equations to calculate the magnitude and phase of the transverse magnetization across the slice profile. Additionally, regional variations of the transmit RF field and slice profile scaling due to macroscopic field gradients were included. Simulations, phantom, and in vivo measurements at 3 T were conducted for $R_{2}^{*}$ and myelin water fraction (MWF) mapping.

**Results:**

The influence of macroscopic field gradients on $R_{2}^{*}$ and myelin water fraction estimation can be substantially reduced by applying the proposed model. Moreover, it was shown that the dephasing over time for flip angles of 60° or greater also depends on the polarity of the slice‐selection gradient because of phase variation along the slice profile.

**Conclusion:**

Substantial improvements in $R_{2}^{*}$ accuracy and myelin water fraction mapping coverage can be achieved using the proposed model if higher flip angles are required. In this context, we demonstrated that the phase along the slice profile and the polarity of the slice‐selection gradient are essential for proper modeling of the gradient‐echo signal in the presence of macroscopic field variations.

## 1 INTRODUCTION

The sensitivity of gradient‐echo (GRE) imaging to variations in magnetic susceptibility has led to a widespread range of applications in MRI. Approaches focusing on the signal decay are methods such as FMRI, in which the susceptibility difference between deoxyhemoglobin and oxyhemoglobin is measured,^1^, ^2^ perfusion MRI with gadolinium‐based contrast agent,^3^ $R_{2}^{*}$ mapping by acquiring multi‐gradient‐echo (mGRE), or the determination of the myelin water fraction (MWF) from analyzing the multi‐exponential decay of GRE signal.^4^ Methods exploiting the phase evolution of the signal are SWI^5^ or QSM.^6^ Quantitative susceptibility mapping and $R_{2}^{*}$ contrast in the brain have been used to study iron content in inflammatory and neurodegenerative diseases.^7^, ^8^, ^9^, ^10^, ^11^

A challenge with quantifying mGRE data are the macroscopic field variations that arise, for example, from air/tissue boundaries, which lead to a faster signal decay and consequently mask tissue‐relevant mesoscopic‐scale and microstructure‐scale $R_{2}^{*}$ effects.^12^ Approaches for correcting the influence of macroscopic field variations can be grouped into methods based on sequence design or postprocessing of conventional mGRE data. Methods requiring sequence adaptations aim to compensate intravoxel dephasing by varying the slice‐rephasing gradient and/or by applying compensation gradients in the slice‐selective direction.^13^, ^14^, ^15^, ^16^, ^17^ Beyond sequence programming, a powerful approach is to simply increase spatial resolution,^18^ which is not always feasible in a clinical setup with scan‐time constraints or limited SNR.

The focus of the present work is the correction of the influence of macroscopic field variations by postprocessing of mGRE data. Given that for 2D‐GRE acquisitions the slice thickness is usually much larger than the in‐plane resolution, signal dephasing is largely driven by macroscopic field variations along the slice‐selective direction *z*.^13^ Assuming an ideal slice profile and a constant field gradient *G_z_* as an approximation of the macroscopic field variation along *z*, the signal over time is sinc‐weighted proportional to *G_z_* and slice thickness.^12^ Based on this theory, Fernandez‐Seara and Wehrli^19^ estimated *G_z_* and $R_{2}^{*}$ iteratively from the measured signal decay, which later was refined by initialization of *G_z_* using the field map^20^ and further extended by Yang et al by modeling field variation as a quadratic function, a condition that has become more relevant at ultrahigh‐field MRI.^21^ Depending on the RF excitation pulse, deviations from the ideal slice profile cause different dephasing in the presence of *G_z_*. To account for various pulse shapes, Preibisch et al proposed a solution in which the signal modulation due to *G_z_* and the slice profile is described by the envelope of the RF pulse.^22^ This model allows to describe the signal decay for flip angles *α* of less than 60° and a much longer TR than the longitudinal relaxation time T_1_ to avoid saturation of the slice profile.^22^ To achieve a smooth signal decay, they have used exponential RF pulses^23^ and compared them with sinc‐shaped and sinc‐Gauss shaped pulses for $R_{2}^{*}$ mapping.^24^

Similar to $R_{2}^{*}$ mapping, in MWF mapping, macroscopic field variations need to be accounted for.^25^ Here, modeling approaches also assume an ideal slice profile.^25^, ^26^ In recent work, Lee et al combined *z*‐shimming with modeling of the magnitude of the slice profile to achieve a better modeling of the signal decay.^27^

In contrast to the analytical solution in Preibisch et al,^22^ which is limited by the small flip angle approximation,^28^ we here propose a numerical model for solving the signal dephasing in the presence of *G_z_* for an arbitrary excitation pulse and flip angle. Consequently, the model allows to benefit from increased SNR in measurements with interleaved slice acquisition, especially for large flip angles (*α* > 60°). Extending the model from Hernando et al,^29^ we also investigate variations of the transmit RF field $B_{1}^{+}$ and the effect of scaling of the slice profile due to superposition of *G_z_* and the slice‐selection gradient *G_slice_*. We further demonstrate with phantom and in vivo measurements that, depending on the pulse shape for larger flip angles, the polarity of *G_slice_* has to be considered, because through‐slice phase variations can severely affect signal dephasing. With the proposed model, it is possible to substantially improve the quality of $R_{2}^{*}$ maps and MWF maps acquired with arbitrary excitation pulses and flip angles.

## 2 METHODS

### 2.1 Theory

In the presence of macroscopic field variations Δ*ω*(*z*), the signal *S*(*t*) of a 2D spoiled GRE is proportional to the integral over the complex transverse magnetization ${\underline{M}}_{\mathrm{xy}}\left(z\right)=\left|M_{\mathrm{xy}}\left(z\right)\right|e^{i\varphi_{\mathrm{xy}}\left(z\right)}$ weighted with Δ*ω*(*z*) along the slice‐selective direction *z*. Thus, depending on ${\underline{M}}_{\mathrm{xy}}\left(z\right)$ and Δ*ω*(*z*), additional signal dephasing is observed in contrast to theoretical mono‐exponential signal decay with $R_{2}^{*}$. If Δ*ω*(*z*) is smooth and slowly varying in space, Δ*ω*(*z*) can be approximated with a linear function $\Delta \omega =\Delta \omega_{0}+\gamma G_{z}z$ in each slice.^12^ By assuming the origin of *z* being in the center of the slice, the equation for *S*(*t*) reads as follows:

(1) $$S\left(t\right)=e^{-R_{2}^{*}t}\int_{-\infty }^{\infty }{\underline{M}}_{\mathrm{xy}}\left(z,\;\alpha \xi ,\;\lambda ,\;E_{1}\right)e^{i\Delta \omega \left(z\right)t}dz\approx e^{-R_{2}^{*}t}\int_{-\infty }^{\infty }{\underline{M}}_{\mathrm{xy}}\left(z,\;\alpha \xi ,\;\lambda ,\;E_{1}\right)e^{i\Delta \omega_{0}t}e^{i\gamma G_{z}zt}dz$$

where *G_z_* denotes the field gradient and Δ*ω*_0_ denotes the field offset. The value of ${\underline{M}}_{\mathrm{xy}}\left(z\right)$ depends on several factors (including ξ, λ, and *E*_1_), discussed in detail subsequently. Depending on the ratio of the TR and the T_1_, which is included in the exponential term $E_{1}={\;e}^{-\text{TR}/T_{1}}$, and the effective flip angle *α_eff_*(*z*) along the slice, the solution for |*M_xy_*(*z*)| changes according to the steady‐state equation for spoiled GRE sequences^30^ as follows:

(2) $$\left|M_{\mathrm{xy}}\left(z,\;\alpha \xi ,\;\lambda ,\;E_{1}\right)\right|=S_{0}\sin\left(\alpha_{\mathrm{eff}}\left(z,\;\alpha \xi ,\;\lambda \right)\right)\frac{1-E_{1}}{1-\cos\left(\alpha_{\mathrm{eff}}\left(z,\;\alpha \xi ,\;\lambda \right)\right)E_{1}}.$$

When TR is much larger than T_1_, Equation 2 simplifies and |*M_xy_*(*z*)| is obtained by the sine of *α_eff_* times the equilibrium magnetization *S*_0_:

(3) $$\left|M_{\mathrm{xy}}\left(z,\;\alpha \xi ,\;\lambda ,\;E_{1}=0\right)\right|=S_{0}\sin\left(\alpha_{\mathrm{eff}}\left(z,\;\alpha \xi ,\;\lambda \right)\right).$$

Here, *α_eff_*(*z*) is obtained for a certain slice‐selection gradient *G_slice_* and the applied excitation pulse with a certain shape and amplitude. For small flip angles, the slice profile *α_eff_*(*z*) can be estimated for an RF pulse envelope *B*_1_(*t*) with the small flip angle approximation.^28^ However, larger flip angles require solving the Bloch equations for |*M_xy_*(*z*)| and *φ_xy_*(*z*).

Extending previous studies, the factors ξ and λ were added to describe 2 effects that affect *α_eff_*(*z*) and therefore signal dephasing. First, variations of the active transmit field ($B_{1}^{+}$) cause a deviation from the nominal flip angle *α*, which can change the effective flip angle profile *α_eff_*(*z*), and therefore requires *α* to be scaled with ξ, obtained from the normalized *B*_1_ map. Second, *G_z_* is superimposed with *G_slice_*, leading to either broadening or narrowing of the slice profile described by the factor λ^31^ as follows:

(4) $$\lambda =\frac{G_{\mathrm{slice}}}{G_{\mathrm{slice}}+G_{z}}.$$

To investigate the effect of the described parameters on signal dephasing in the presence of *G_z_*, 4 different models have been studied. Summarizing Equation 1 in a tissue‐specific signal component *S*_tissue_(*t*) (e.g., $S_{\text{tissue}}\left(t\right)=S_{0}e^{-R_{2}^{*}t}$) and a component *F_i_*(*t*) describing the signal dephasing due to Δ*ω*(*z*), the model $S_{i}\left(t\right)$ can be written as $S_{i}\left(t\right)=S_{\text{tissue}}\left(t\right)F_{i}\left(t\right)$. The 4 models are defined as follows:

(5) $$S_{1}\left(t\right)=S_{\text{tissue}}\left(t\right)\;F_{1}\left(t\right)=S_{\text{tissue}}\left(t\right)$$

(6) $$S_{2}\left(t\right)=S_{\text{tissue}}\left(t\right)\;F_{2}\left(t\right)=S_{\text{tissue}}\left(t\right)\int_{-\infty }^{\infty }\left|M_{\mathrm{xy}}\left(z,\;\alpha ,\;\lambda =1,\;E_{1}=0\right)\right|e^{{i\gamma G}_{z}\mathrm{zt}}\mathrm{dz}$$

(7) $$S_{3}\left(t\right)=S_{\text{tissue}}\left(t\right)\;F_{3}\left(t\right)=S_{\mathrm{tissue}}\left(t\right)\int_{-\infty }^{\infty }\left|M_{\mathrm{xy}}\left(z,\;\alpha ,\;\lambda =1,\;E_{1}=0\right)\right|e^{{i\varphi }_{\mathrm{xy}}\left(z,\;\alpha ,\;\lambda =1,\;E_{1}=0\right)}e^{{i\gamma G}_{z}\mathrm{zt}}\mathrm{dz}$$

(8) $$S_{4}\left(t\right)=S_{\text{tissue}}\left(t\right)\;F_{4}\left(t\right)=S_{\text{tissue}}\left(t\right)\int_{-\infty }^{\infty }\left|M_{\mathrm{xy}}\left(z,\;\alpha \xi ,\;\lambda ,\;E_{1}=0\right)\right|e^{i\varphi_{\mathrm{xy}}\left(z,\;\alpha \xi ,\;\lambda ,\;E_{1}=0\right)}e^{{i\gamma G}_{z}\mathrm{zt}}\mathrm{dz}$$

The model *S*_1_(*t*) serves as an uncorrected reference without modeling *M*_*xy*_(*z*) and Δ*ω*(*z*). Then, for *S*_2_(*t*), only the magnitude along the slice |*M_xy_*(*z*)| was considered neglecting *φ_xy_*(*z*). In *S*_3_(*t*), *φ_xy_*(*z*) was included, and in *S*_4_(*t*) the model was extended by additionally incorporating $B_{1}^{+}$ and λ variations.

### 2.2 Numerical implementation

Signal dephasing due to *G_z_* was estimated numerically for F_2_ to F_4_ assuming *E*_1_ = 0. In the first step, *M*_*xy*_ was estimated for a certain RF excitation pulse and *G_slice_* with a freely available numerical Bloch solver using *MATLAB* (MathWorks, Natick, MA).^32^ Simulations were carried out with temporal resolution of 2 µs and spatial resolution of 80 µm with 2501 spatial points. The normalized envelope *B*_1_(*t*) was scaled to achieve *α_eff_*(*z* = 0) = *α*ξ in the center of the slice. Rather than estimating *M*_*xy*_ for each voxel with ξ and λ, calculations were accelerated by estimating *M*_*xy*_ in steps of Δξ = 0.05 followed by linear interpolation to Δξ_intp_ = 0.005. Variations of λ were incorporated by multiplying the sampling points along *z* with λ, to scale the thickness of the slice. In the last step, the integral along *z* for given *G_z_* was solved by numerical integration. The source code can be found at: https://github.com/neuroimaging-mug. 

### 2.3 Simulations

To investigate the influence of the *G_slice_* polarity and flip angle *α* on F_3_, simulations for *α* = 30° and *α* = 90° with negative and positive polarity of *G_slice_* were performed. Based on the vendor’s standard GRE pulse, a sinc‐Hanning‐windowed excitation pulse with a pulse duration T_pulse_ of 2 ms and a bandwidth time (BWT) product of 2.7 was chosen for the experiments. A *G_slice_* of 8.29 mT/m was determined with the Bloch solver to achieve a slice thickness ∆z of 4 mm, as defined by the FWHM of |*M_xy_*| for *α* = 30°. Based on the observed field gradients in phantom measurements, *G_z_* was set to 100 µT/m for all simulations. In in vivo measurements of the brain, field gradients up to 300 µT/m have been reported in areas such as orbitofrontal cortex or inferior temporal lobe.^33^

Exploiting the relevance of individual parameters for modeling F_4_, a sensitivity analysis was performed for *φ_xy_*, $B_{1}^{+}$, and λ with the same sinc‐Hanning‐windowed excitation pulse. To estimate the relevance of *φ_xy_*, simulations with *G_z_* = 100 µT/m were carried out for F_4_ with varying *α* from 10° to 90°, each with positive and negative *G_slice_* polarity. Results were compared with simulations for model F_2_ considering only the magnitude |*M_xy_*| of the slice profile (*φ_xy_* = 0). For evaluation, the RMS error (RMSE) over time for each *α* between F_4_ and F_2_ was calculated.

The sensitivity for $B_{1}^{+}$ was simulated by scaling $B_{1}^{+}$ for each flip angle (*α* = 30° and *α* = 90°) with a factor ξ (ranging from 0.6 to 1.4) for *G_z_* = 100 µT/m. The results for F_4_ obtained for different ξ were compared with those for ξ = 1 by plotting the RMSE. Same steps as for $B_{1}^{+}$ were carried out for λ by changing the value from 0.8 to 1.2.

A crucial assumption with the proposed models is that for a given *α*, TR is long enough to avoid changes of |*M_xy_*| due to incomplete T_1_ relaxation $\left(E_{1}=e^{-\text{TR}/T_{1}}\neq 0\right)$. Hence, the steady‐state solution in Equation 2 was included to estimate signal dephasing F_T1_ in the presence of *G_z_* = 100 µT/m for different *E*_1_:

(9) $$F_{T1}\left(t\right)=\int_{-\infty }^{\infty }\left|M_{\mathrm{xy}}\left(z,\;\alpha \xi ,\;\lambda ,\;E_{1}\right)\right|e^{i\varphi_{\mathrm{xy}}\left(z,\;\alpha \xi ,\;\lambda ,\;E_{1}\right)}e^{i\gamma G_{z}zt}dz.$$

For each TR/T_1_ (ranging from 1 to 5), the Ernst‐angle *α_Ernst_* was calculated and simulations with the sinc‐Hanning‐windowed RF pulse (T_pulse_ = 2 ms and BWT = 2.7) were carried out by setting *α* = *α_Ernst_*, *α* = 0.8 *α_Ernst_*, and *α* = 0.6 *α_Ernst_*. Obtained results were compared by calculating the RMSE over time between F_T1_ and F_3_.

### 2.4 Phantom experiments

To validate the results from the simulations of dephasing effects for different *α* and *G_slice_*, polarity phantom measurements were performed. For the phantom, a plastic cylinder (Ø = 12 cm and length = 20 cm) was filled with agarose gel (5 g/L), which was doped with 110 µmol/L MAGNEVIST to shorten the T_1_.

The phantom was scanned on a 3 T MRI system (Magnetom Prisma; Siemens, Erlangen, Germany) twice by a mGRE sequence with *α* = 30° and *α* = 90°, each with alternating polarity of *G_slice_*. The same sinc‐Hanning‐windowed excitation pulse (T_pulse_ = 2 ms and BWT = 2.7) as for the simulations was used, and |*G_slice_*| = 8.29 mT/m was used to achieve ∆*z* = 4 mm for *α* = 30°.

Other sequence parameters were as follows: FOV = 128 × 128 mm^2^, in‐plane resolution = 1 × 1 mm^2^, 32 monopolar echoes with bandwidth = 500 Hz/px, TE_1_ = 4 ms, ΔTE = 5 ms, TR = 3 seconds, 25 slices, with 0% interslice gap. For *B*_1_ mapping, a Bloch‐Siegert sequence with the same resolution was used.^34^

The *G_z_* map was obtained by using the central difference from the field map Δ*B*_0_ to estimate the gradient in the *i*th slice:

(10) $$G_{z}\left(x,\;y,\;z_{i}\right)=0.5\frac{\Delta B_{0}\left(x,\;y,\;z_{i+1}\right)-\Delta B_{0}\left(x,\;y,\;z_{i-1}\right)}{\Delta z}.$$

Single side difference was used for the first (*i* = 1) and last slice (*i* = *N*). The value of Δ*B*_0_ was estimated from a linear fit of the first 6 echoes of the unwrapped phase (PRELUDE unwrapping^35^). From the measured data, $R_{2}^{*}$ maps were estimated in *MATLAB* using the lsqnonlin() function with models *S*_1_ to *S*_4_.

As indicated in Supporting Information Figure S1, when varying *G_slice_* amplitude slightly within the model, it was found that results could be further improved when using *G_slice_* = 8.5 mT/m for all analyses.

### 2.5 Influence of TR/T_1_

Phantom measurements with different TRs (125 ms, 250 ms, 500 ms, 1 second, 1.5 seconds, 2 seconds, 3 seconds, and 5 seconds) and *α* (30°, 60°, and 90°) were carried out with the mGRE sequence to investigate steady‐state effects for modeling. A Bloch‐Siegert sequence was used for *B*_1_ mapping.^34^ In addition, T_1_ was estimated with an inversion recovery sequence with 6 TIs (100 ms, 200 ms, 400 ms, 800 ms, 1.6 seconds, and 3.6 seconds), and the excited slice was measured with 0.5 × 0.5 mm^2^ in‐plane resolution by changing the readout direction to the slice direction. Results were evaluated by estimating $R_{2}^{*}$ with model *S*_4_ for each TR and *α*.

### 2.6 In vivo $R_{2}^{*}$ and MWF experiments

To evaluate the proposed modeling for in vivo application, $R_{2}^{*}$ and MWF mapping experiments were performed on the same 3 T MRI system with 10 subjects (age range = 26‐50 years). The study was approved by the local ethics committee, and all subjects gave written informed consent. In addition, subjects were scanned with an anatomical MPRAGE with 1‐mm^3^ isotropic resolution for regional evaluation of $R_{2}^{*}$ and MWF maps.

For $R_{2}^{*}$ mapping, subjects were scanned twice with a mGRE sequence with alternating *G_slice_* polarity using a sinc‐Hanning‐windowed excitation pulse (T_pulse_ = 2 ms and BWT = 2.7) with *α* = 85° (Ernst angle assuming T_1_ = 1 second). Other sequence parameters were as follows: FOV = 256 × 208 mm^2^, in‐plane resolution = 1 × 1 mm^2^, |*G_slice_*| = 11.05 mT/m to achieve ∆z = 3 mm, 17 monopolar echoes with bandwidth = 500 Hz/px, TE_1_ = 2.87 ms, ΔTE = 3.59 ms, TR = 2.5 seconds, and 30 slices with 0% interslice gap. The last echo was a navigator echo at TE_navi_ = 65.4 ms, to correct for physiologically induced field variations.^36^ Then for each channel, the *n*th phase‐encoding line $S_{n}\left(k_{x},\;TE\right)$ was corrected as described by Wen et al^37^:

(11) $$S_{n}^{c}\left(k_{x},\;TE\right)=S_{n}\left(k_{x},\;TE\right)\exp\left(-i\frac{\phi_{n}-\phi_{1}}{TE_{\mathrm{navi}}}TE\right),$$

where *ϕ_n_* and *ϕ*_1_ are the mean phase values of the *n*th navigator echo and the reference phase of the first navigator echo, respectively. To account for phase accumulation after excitation, the estimated phase difference was scaled with the TE. Afterward, corrected k‐space data were combined with the method proposed in Luo et al.^38^

For *B*_1_ mapping, a highly accelerated method based on the Bloch‐Siegert shift was used.^39^ The field map for calculating *G_z_* was obtained from the difference of the unwrapped phase of the first and third echo divided by TE difference. From the data, $R_{2}^{*}$ maps were obtained using the models *S*_1_, *S*_3_, and *S*_4_. The difference between the models was assessed regionally by calculating the mean and SD of $R_{2}^{*}$ in all subjects in gray matter and global white‐matter masks. Gray matter masks were segmented from the MPRAGE images with FSL FIRST,^40^ and the global white matter masks with SIENAX,^41^ part of FSL.^42^ All masks were affinely registered to mGRE space with FSL FLIRT.^43^, ^44^

For MWF mapping, all subjects were scanned with a slightly adapted mGRE sequence to account for the fast decaying myelin water component. Short echo spacing (ΔTE = 2.2 ms) was achieved with a bipolar readout gradient, which was inverted in a second acquisition to compensate for phase errors between even and odd echoes. Other sequence parameters were as follows: sinc‐Hanning‐windowed excitation pulse with T_pulse_ = 1 ms and BWT = 2, *α* = 85°, *G_slice_* = 14.15 mT/m, FOV = 255 × 105 mm^2^, in‐plane resolution = 1.14 × 1.14 mm^2^, ∆*z* = 4 mm, 27 bipolar echoes with bandwidth = 500 Hz/Px, TE_1_ = 2.37 ms, ΔTE = 2.2 ms, TR = 2 seconds, TE_navi_ = 63.8 ms, 25 interleaved slices with 0% interslice gap, and total scan time = 12 minutes. Again, a highly accelerated *B*_1_ map was acquired.^39^

After correction of the data with the navigator echoes, the 2 mGRE images were registered using FSL FLIRT^45^ before averaging. The MWF estimation was based on a multi‐exponential $T_{2}^{*}$ relaxation times model^46^ with M = 200 water components:

(12) $$S_{\text{tissue}}\left(\mathrm{TE}\right)=\sum_{j=1}^{M}s_{j}\exp\left(-\frac{\mathrm{TE}}{T_{2,j}^{*}}\right).$$

Evaluation of data was performed by estimating MWF maps using models *S*_1_, *S*_3_, and *S*_4_ with the nonnegative least squares algorithm of the MERA toolbox^47^ and a cutoff for myelin water $T_{2}^{*}$_my_ < 25 ms.^48^ For *S*_3_ and *S*_4_, the measured signal S was corrected with F_3_ and F_4_, respectively, before parameter estimation.

Regional evaluation of MWF maps was performed in white matter tracts with the JHU white‐matter atlas.^49^ The atlas was nonlinearly registered with FSL FNIRT to the MPRAGE images and transformed to the mGRE space using FSL FLIRT.^43^, ^44^ Before evaluation, masks were manually checked and adjusted with ITK‐SNAP.^50^

In a single scan session, 8 mGRE data sets were acquired from 1 subject (male, age = 29) using 4 different excitation pulses with *α* = 30° and 85° for each pulse. The first pulse was a 2‐ms‐long Gaussian pulse with *σ* = 280 µs ($B_{1}\left(t\right)=e^{-\frac{t}{2\sigma }}$), and the other 3 were sinc‐Hanning‐windowed pulses with different BWT = 2, 2.7, and 8 and T_pulse_ = 1 ms, 2 ms, and 4 ms. The value of *G_slice_* = 10.56 mT/m, 18.87 mT/m, 11.05 mT/m, and 15.65 mT/m was estimated with the Bloch solver for ∆*z* = 3 mm and *α* = 30°. Other sequence parameters, as well as *B*_1_ mapping, were as described for in vivo $R_{2}^{*}$ mapping. The differences between the pulses were assessed by estimating $R_{2}^{*}$ maps with *S*_4_.

## 3 RESULTS

### 3.1 Simulations

**Figure 1:**
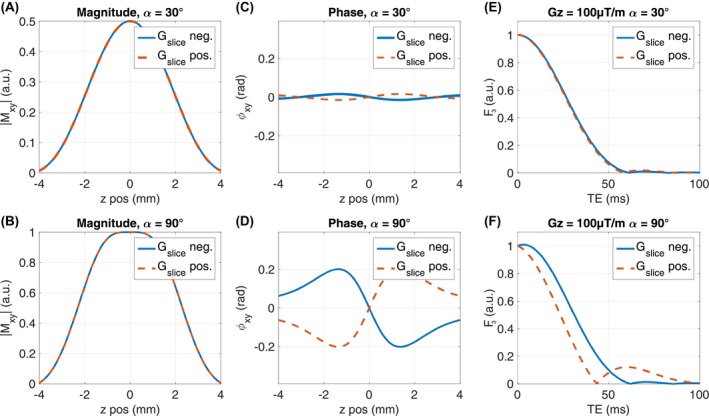
Simulation results for magnitude |*M_xy_*| (A,B) and phase *φ_xy_* (C,D) of the slice profile and the resulting dephasing F_3_ (E,F) with a macroscopic field gradient *G_z_* = 100 µT/m for a sinc‐Hanning‐windowed excitation pulse (pulse duration T_pulse_ = 2 ms, bandwidth time product BWT = 2.7). For each *α* (top *α* = 30*°*, bottom *α* = 90*°*), simulations were performed with positive (red dotted line) and negative (solid blue line) *G_slice_* polarity. There is no difference in the magnitude (A,B) but the mirrored phase for *α* = 90*°* (D) causes different F_3_ (F)

Figure 1 shows the simulation results for sinc‐Hanning‐windowed excitation pulse with positive and negative *G_slice_* polarity for *α* = 30° and *α* = 90°. It reveals that the polarity has no influence on |*M_xy_*(*z*)| of the slice profile (Figure 1A,B), whereas *φ_xy_*(*z*) is inverted when flipping polarity (Figure 1C,D). Consequently, F_3_ depends on the polarity of *G_slice_* (Figure 1E,F), an effect that is more strongly pronounced for *α* = 90°.

**Figure 2:**
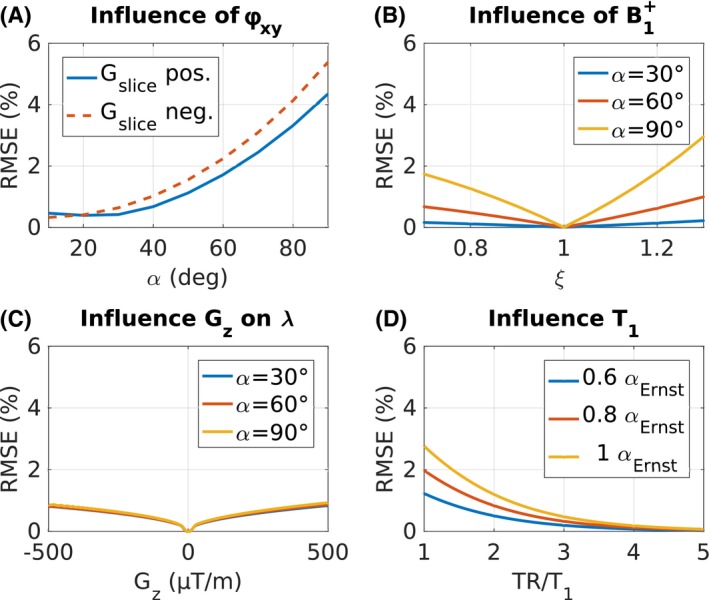
Sensitivity analysis of the numerical model parameters. A, Comparison of the effect of including phase φ_xy_ in F_4_ versus a magnitude model F_2_ for positive and negative *G_slice_* polarity and *G_z_* = 100 µT/m. B, Effects of $B_{1}^{+}$ variations in F_4_ with a macroscopic field gradient *G_z_* = 100 µT/m. C, Influence of *G_z_* on the slice encoding described with λ. D, The RMS error (RMSE) for neglecting T_1_ for different TR/T_1_ ratios is plotted assuming *G_z_* = 100 µT/m. For each TR/T_1_, the RMSE was estimated between the F_4_ and F_T1_ for *α_Ernst_*, 0.8 *α_Ernst_*, and 0.6 *α_Ernst_*

The sensitivities of the model parameters *φ_xy_*(*z*), $B_{1}^{+}$, λ, and TR/T_1_ are illustrated in Figure 2. When neglecting *φ_xy_*(*z*) in Figure 2A, the RMSE substantially increases for *α* > 40° with larger RMSE for negative *G_slice_*. For *α* = 90°, the RMSE is 5.5% for negative polarity and 4.5% for positive polarity, respectively.

The sensitivity for $B_{1}^{+}$ variations in Figure 2B depends strongly on the nominal flip angle *α*. For *α* = 30°, the RMSE was below 0.5% for all simulated values of ξ (*α_effective_* = α*ξ), with a moderate increase for *α* = 60° to 1% for ξ = 1.3. With 2.9%, the RMSE was 3 times higher for *α* = 90°.

The influence of λ on the signal is relatively small compared with $B_{1}^{+}$ and *φ_xy_*(*z*) with an RMSE of 0.8% for a strong *G_z_* with 500 µT/m and minimal dependency on *α* (Figure 2C).

The simulated error due to neglecting T_1_ for different TR/T_1_ in Figure 2D shows an exponential decrease of the RMSE with increasing TR/T_1_ for all simulated flip angles. For all TR/T_1_ ratios, the highest RMSE was estimated when using the Ernst‐angle *α_Ernst_* and declines nonlinearly for 0.8 *α_Ernst_* and 0.6 *α_Ernst_*. For example, for TR/T_1_ = 1, the RMSE decreases from 2.8% to 1.8% to 1.2% for all simulated flip angles, whereas for TR/T_1_ = 2 the RMSE reduces from 1.2% to 0.8% to 0.5%.

When comparing the simulated errors by neglecting *φ_xy_* in Figure 2A with T_1_ effects in Figure 2D, the RMSE of *φ_xy_* becomes dominant with increasing TR/T_1_ ratio. Given that TR/T_1_ > 2, which results in *α_Ernst_* > 82°, the RMSE is smaller than 1.2%, whereas the RMSE due to neglecting *φ_xy_* is at least higher than 3.3% depending on the *G_slice_* polarity.

### 3.2 Phantom experiments

**Figure 3:**
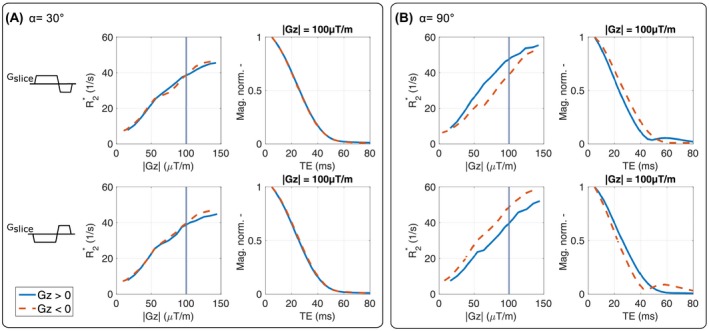
Comparison of $R_{2}^{*}$ values estimated from the phantom experiments with the mono‐exponential model *S*_1_ are plotted as function of *G_z_* for *α* = 30*°* (A) and *α* = 90*°* (B) with positive and negative slice‐selection gradient *G_slice_*. Additionally, the averaged normalized signal decay is plotted for |*G_z_*| = 100 µT/m. The dotted red line represents a positive *G_z_* and the solid blue line represents a negative *G_z_*. For *α* = 30*°*, no relevant differences between the polarity of *G_slice_* and *G_z_* are observed, whereas for *α* = 90*°*, flipped *G_slice_* polarity substantially affects $R_{2}^{*}$

The $R_{2}^{*}$ values estimated with the mono‐exponential model *S*_1_ are plotted as a function of *G_z_* for *α* = 30° and *α* = 90° with positive and negative *G_slice_* polarity in Figure 3. The value of $R_{2}^{*}$ increases proportional to *G_z_* for *α* = 30° (Figure 3A) with up to 8‐times higher $R_{2}^{*}$ values for *G_z_* = 150 µT/m than for *G_z_* = 0 µT/m. For *α* = 30°, negligibly small differences between the polarity of *G_slice_* and the sign of *G_z_* were found, whereas for *α* = 90° (Figure 3B), positive and negative *G_z_* yield different $R_{2}^{*}$ values and a dependency on the polarity of *G_slice_*. Moreover, Figure 3 shows the normalized averaged signal decay for |*G_z_*| = 100 µT/m plotted with positive and negative *G_z_*, explaining the difference in estimated $R_{2}^{*}$ values. For *α* = 90° with positive *G_slice_* and *G_z_* > 0 (blue line), the signal decays faster than for *G_z_* < 0 (red) and vice versa when switching *G_slice_* polarity.

**Figure 4:**
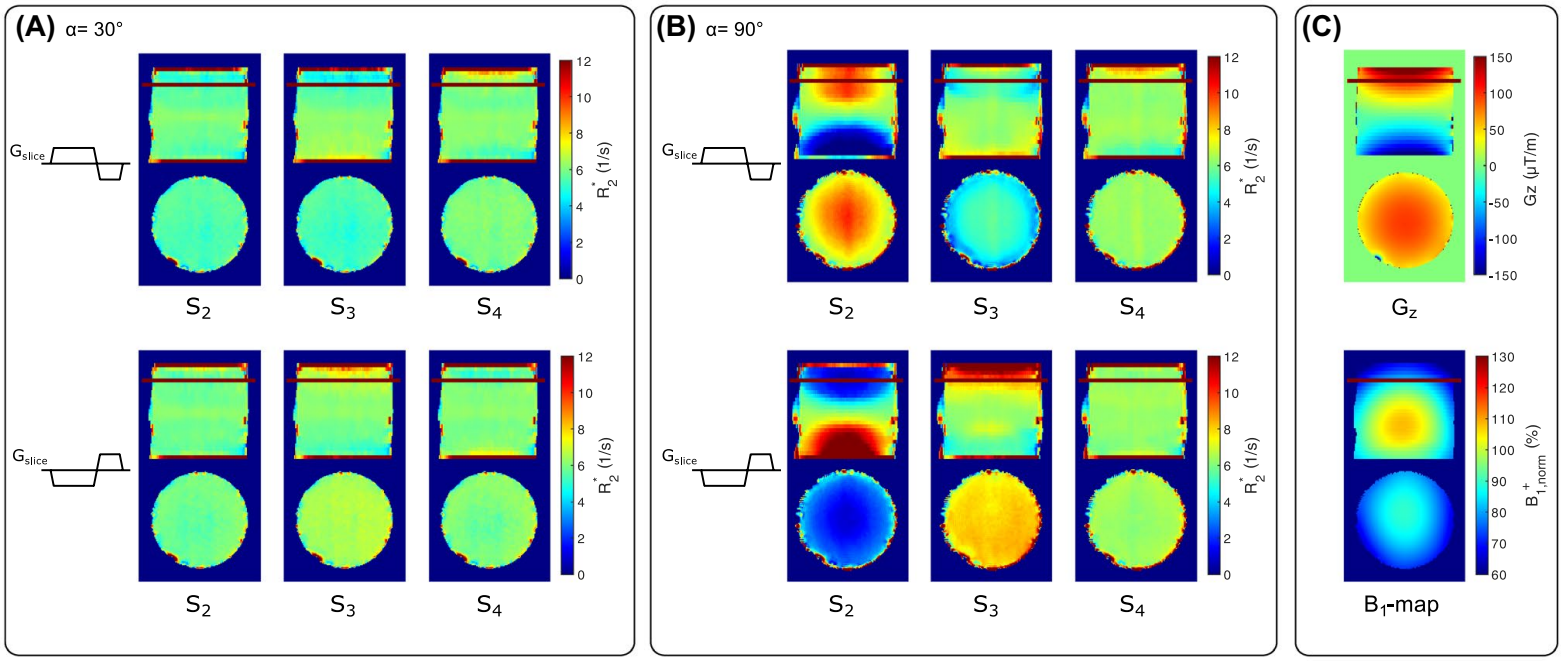
Coronal and axial slices of estimated $R_{2}^{*}$ maps from the phantom measurements for different signal models (S_2_ ‐S_4_). Although all correction models yield relatively comparable $R_{2}^{*}$ values for *α* = 30*°* (A), the high flip angle results for *α* = 90*°* (B) highlight the effect of $B_{1}^{+}$ and λ correction. Full modeling with *S*_4_ also eliminates the influence of the polarity of the slice‐selection gradient *G_slice_* at *α* = 90*°*. The corresponding *G_z_* maps and *B*_1_ maps are shown in (C)

Figure 4 compares the $R_{2}^{*}$ maps obtained from fits using models *S*_2_, *S*_3_, and *S*_4_ for *α* = 30° (Figure 4A) and *α* = 90° (Figure 4B), each with positive and negative *G_slice_* polarity. In addition, the *G_z_* map and *B*_1_ map are illustrated in Figure 4C. Although results for *α* = 30° are comparable for all models, considerable differences for *α* = 90° between models and *G_slice_* polarity were found. When using only the magnitude |*M_xy_*| in model *S*_2_ to estimate $R_{2}^{*}$ for *α* = 90°, it was not possible to recover $R_{2}^{*}$ without the influence of *G_z_*. The $R_{2}^{*}$ values for *G_z_* > 0 were overestimated for positive *G_slice_* and underestimated for *G_z_* < 0, and switching to negative *G_slice_* polarity inverted the results. Extending the model *S*_2_ by adding *φ_xy_* in *S*_3_ yields better maps, which are influenced less by the *G_slice_* polarity. Additionally, including $B_{1}^{+}$ and λ in *S*_4_ substantially improves $R_{2}^{*}$ maps, with minimal differences between *G_slice_* polarities. Further, estimated $R_{2}^{*}$ maps using *S*_4_ with *α* = 90° are comparable with maps estimated from *α* = 30° for both *G_slice_* polarities.

**Figure 5:**
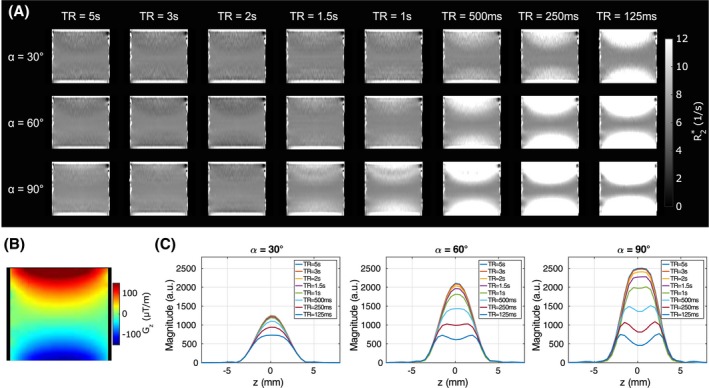
Experimental evaluation of TR/T_1_ dependency for $R_{2}^{*}$ modeling in phantom measurements. A, Coronal $R_{2}^{*}$ maps were estimated using *S*_4_ for different TR and *α*. The minimum TR required for avoiding T_1_ effects increases with the magnitude of *G_z_* (B) and *α*. The value of T_1_ = 740 ± 86 ms was estimated with an inversion‐recovery sequence. C, The measured signal along the slice for each *α* and TR shows the different steady‐state solutions

Figure 5 illustrates the effects of neglecting T_1_ for signal modeling. Estimated $R_{2}^{*}$ maps with *S*_4_ (Figure 5A) indicate an overestimation of $R_{2}^{*}$, depending on TR and *α* in the presence of *G_z_* (Figure 5B). For *α* = 30°, increased $R_{2}^{*}$ values are observable only up to a TR of 500 ms, whereas for *α* = 90° these effects extend up to a TR of 1.5 seconds. These TR values correspond to a TR/T_1_ ratio of 0.67 and 2.01 for the estimated T_1_ = 740 ms. The origin for the $R_{2}^{*}$ overestimation is shown in Figure 5C, where the averaged measured signal along the slice profile is plotted. Depending on *α* and TR, the steady‐state solution changes, causing a modeling error in the presence of *G_z_*. Between different TRs for *α* = 30°, the profiles show less variations compared with *α* = 90°, leading to different signal dephasing for the same *G_z_*. In addition to T_1_ effects, for TR > 2 seconds, SNR benefits can be observed for maps acquired with *α* = 90° compared with *α* = 30°.

### 3.3 In vivo experiments

**Figure 6:**
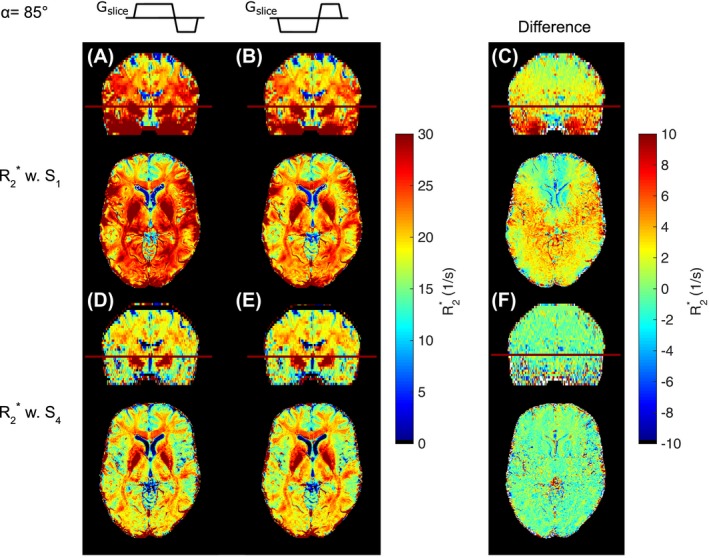
Comparison of coronal and axial $R_{2}^{*}$ maps obtained from mono‐exponential model *S*_1_ (A,B) with maps from the proposed numerical model *S*_4_ (D,E) for positive and negative slice‐selection gradient *G_slice_*. C,F, Difference map between *G_slice_* polarities for each model. The *S*_1_ model shows $R_{2}^{*}$ overestimation and substantial impact of the *G_slice_* polarity (C), which were mitigated using *S*_4_ (F)

In vivo results of $R_{2}^{*}$ maps obtained with models *S*_1_ and *S*_4_ are illustrated in Figure 6 for both *G_slice_* polarities. When comparing *S*_1_ (Figure 6A,B) with *S*_4_ (Figure 6D,E), much higher $R_{2}^{*}$ values are observed in maps using *S*_1_ compared with *S*_4_, thereby minimizing the effects of *G_z_*. In addition, the difference map between positive and negative *G_slice_* polarity for each model reveals strong variations of $R_{2}^{*}$ values with up to 10 s^−1^ for *S*_1_ in areas with strong *G_z_* (Figure 6C). In contrast, maps estimated with *S*_4_ substantially suppressed the effect of *G_slice_* polarity with difference values below 1 s^−1^ (Figure 6F).

In Table 1 the regional evaluation of $R_{2}^{*}$ values with the corresponding mean |*G_z_*| across all subjects is presented. Compared with the other models, the highest $R_{2}^{*}$ values were obtained with *S*_1_ in all anatomical regions. In addition, the difference between *G_slice_* polarities increases with the mean |*G_z_*| value in each region for *S*_1_. For example, in the caudate nucleus, where the smallest |*G_z_*| was observed with 20 µT/m, the difference between polarities is below 0.1 s^−1^, whereas in the brainstem it is 7.46 s^−1^ at a mean |*G_z_*| of 89 µT/m. The $R_{2}^{*}$ values generally decrease when using *S*_2_, but the difference between polarities slightly increases compared with *S*_1_. Applying models *S*_3_ and *S*_4_ reduces the discrepancy between *G_slice_* polarities to a maximum of 2.01 s^−1^ and 1.25 s^−1^ in the brainstem. In all other regions the difference is much smaller, with values below 0.8 s^−1^. Between models *S*_3_ and *S*_4_, rather small changes can be observed generally.

**Table 1:** $R_{2}^{*}$ values (s^−1^) from models *S*_1_ to *S*_4_ in different brain regions for 10 subjects with the corresponding |*G_z_*| values for positive and negative *G_slice_*

Region	*G_slice_*	*S*_1_	*S*_2_	*S*_3_	*S*_4_	|*G*_z_| (µT/m)
Global WM	pos.	26.34 (1.16)	21.11 (0.61)	20.10 (0.58)	19.63 (0.62)	43.06 (8.81)
	neg.	23.72 (0.97)	18.17 (0.58)	19.89 (0.54)	20.17 (0.50)	43.68 (8.40)
Caudate Nucleus	pos.	23.36 (1.53)	21.46 (1.47)	21.81 (1.40)	21.82 (1.40)	20.47 (3.57)
	neg.	23.41 (1.61)	21.58 (1.32)	21.58 (1.28)	21.52 (1.27)	20.42 (3.22)
Pallidum	pos.	39.83 (2.78)	36.56 (2.58)	35.60 (2.65)	34.85 (2.71)	34.38 (8.75)
	neg.	36.86 (2.75)	33.50 (3.08)	35.07 (2.85)	35.61 (2.81)	34.03 (8.73)
Putamen	pos.	29.11 (2.14)	25.78 (1.70)	25.02 (1.76)	24.49 (1.80)	32.69 (5.84)
	neg.	26.97 (2.06)	23.52 (2.05)	24.91 (1.90)	25.28 (1.87)	32.90 (5.87)
Thalamus	pos.	25.84 (1.80)	22.34 (0.64)	21.33 (0.62)	20.34 (0.84)	33.65 (8.75)
	neg.	22.61 (0.97)	18.80 (1.24)	20.50 (0.87)	21.22 (0.74)	34.41 (8.83)
Brainstem	pos.	35.15 (7.97)	20.34 (2.07)	17.58 (1.77)	15.10 (1.60)	88.61 (35.73)
	neg.	27.70 (6.99)	11.21 (1.96)	15.08 (1.53)	16.45 (1.55)	89.90 (34.79)

Note

The $R_{2}^{*}$ and |*G_z_*| values are shown as mean (SD).

Abbreviations: neg., negative; pos., positive; and WM, white matter.

**Figure 7:**
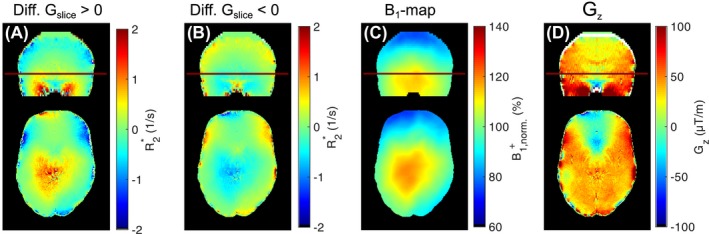
Difference between $R_{2}^{*}$ maps estimated with *S*_4_ (includes $B_{1}^{+}$ and λ variations) and *S*_3_ for positive (A) and negative slice‐selection gradient *G_slice_* (B). Coronal (upper row) and axial (lower row) views are shown. C, *B*_1_ map. D, *G_z_* map. Depending on *G_slice_* polarity, $R_{2}^{*}$ varies in areas with higher $B_{1}^{+}$ and *G_z_* variations

The difference between $R_{2}^{*}$ estimation with *S*_4_ and *S*_3_ is shown in Figure 7, pointing out the effect of modeling $B_{1}^{+}$ and λ in *S*_4_. When visually comparing the difference maps in Figure 7A,B, a strong correspondence between the magnitude of *G_z_* (Figure 7C) and $B_{1}^{+}$ (Figure 7D) can be observed for both *G_slice_* polarities.

**Figure 8:**
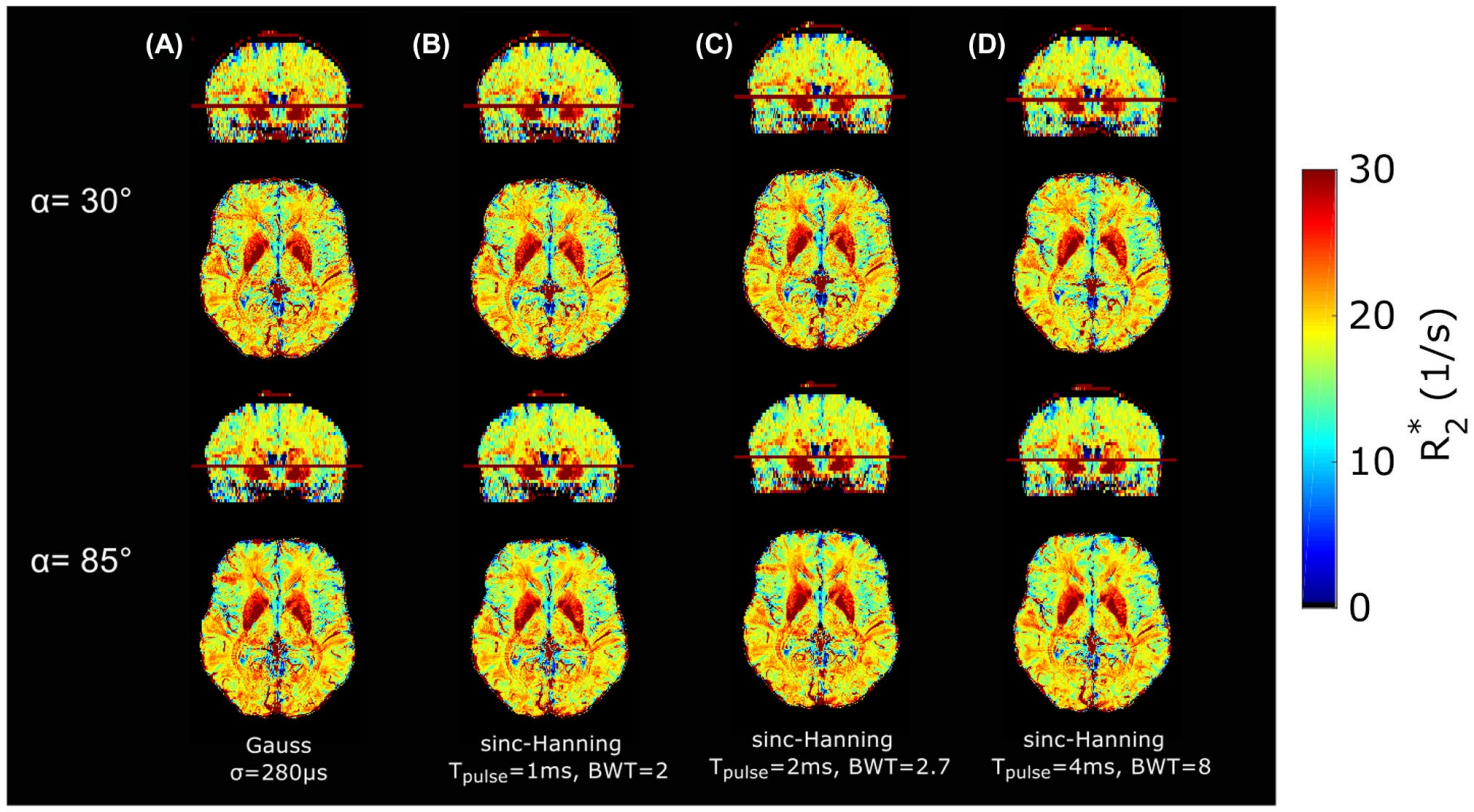
$R_{2}^{*}$ maps estimated with model *S*_4_ from multi‐gradient‐echo (mGRE) data acquired with 4 different excitation pulses (A‐D) for *α* = 30° (top row) and *α* = 85° (bottom row). Regional evaluation of $R_{2}^{*}$ can be found in Supporting Information Table S1

The $R_{2}^{*}$ maps from data acquired with 4 different excitation pulses and 2 different flip angles are shown in Figure 8. Visually, only minor differences among all maps are observable. Higher SNR can be observed in maps with *α* = 85° compared with *α* = 30°. Mean regional $R_{2}^{*}$ values are in good agreement after applying models *S*_3_ and *S*_4_ (Supporting Information Table S1). For example, in global white matter, the largest SD of $R_{2}^{*}$ between the acquisitions was found for *S*_1_ with 1.59 s^−1^, due to the different pulses and flip angles. By using *S*_2_, it decreases to 0.82 s^−1^, and for *S*_3_ and *S*_4_ the estimated values are 0.19 s^−1^ and 0.2 s^−1^, respectively.

**Figure 9:**
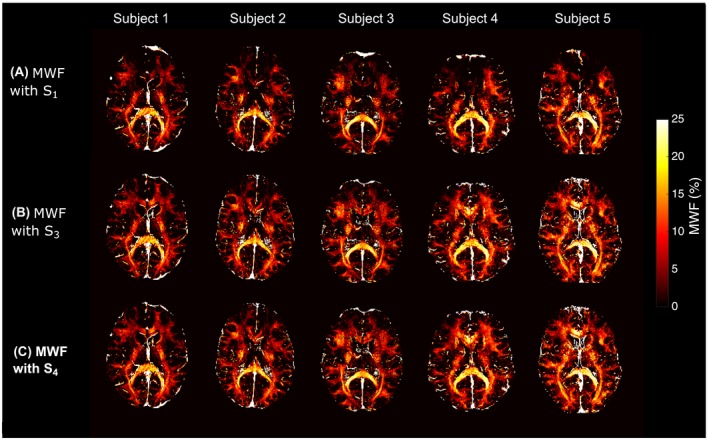
Representative myelin water fraction (MWF) maps from 5 subjects, obtained using models *S*_1_ (A), *S*_3_ (B), and *S*_4_ (C). The proposed models *S*_3_ and *S*_4_ allow us to recover MWF values in areas strongly affected by the field gradient *G_z_* (e.g., in frontal areas)

Figure 9 shows representative slices of MWF maps from 5 subjects obtained with models *S*_1_, *S*_3_, and *S*_4_. It shows that with *S*_1_, in areas with strong *G_z_*, such as in the frontal and temporal lobe, the MWF estimation was not feasible, whereas the proposed approaches allowed a reconstruction in these areas. Between maps with models *S*_3_ and *S*_4_, no considerable differences were found, indicating that $B_{1}^{+}$ and λ have a neglectable small influence.

As shown in Figure 9, the MWF in the genu of the corpus callosum is underestimated with *S*_1_ because of *G_z_*. Using *S*_3_ and *S*_4_ enabled us to recover MWF values in these areas with a median of 12.09% and 12.66%, respectively. Our MWF results are within the range of reported values: For the genu of the corpus callosum, Lee et al^27^ reported approximately 12% for their postprocessing approach, and Alonso‐Ortiz et al^26^ reported approximately 16%. Furthermore, in the body of the corpus callosum, the proposed models yield to an increase of MWF from 3.7% with *S*_1_ to 6.65% and 6.67% for *S*_3_ and *S*_4_, respectively. Interestingly, this analysis demonstrated that rather small |*G_z_*| with around 10 µT/m in the body of the corpus callosum severely affects MWF estimation when using the simple model *S*_1_. Supporting Information Table S2 summarizes the median MWF values in all 10 subjects in different white‐matter regions for models *S*_1_, *S*_3_, and *S*_4_.

## 4 DISCUSSION

In this work we have introduced a numerical model for the signal dephasing of 2D mGRE sequences for arbitrary excitation pulses in the presence of a macroscopic field gradient *G_z_*. In contrast to existing analytical solutions, our model is based on solving the Bloch equations numerically, which allows to estimate signal dephasing for any given flip angle *α*. We have shown that it is indispensable to consider the phase along the slice profile *φ_xy_* and the polarity of the slice‐selection gradient *G_slice_* for describing the signal dephasing for higher *α*. In our experiments, the threshold was approximately 60°, but this may also vary with the RF‐pulse shape.

Compared with existing models,^19^, ^20^, ^22^, ^23^, ^24^, ^26^, ^27^ which include the slice profile and assume linear varying macroscopic field variations, with the proposed model it is possible to explain different signal decays for different signs of *G_z_* observed when using larger flip angles. As illustrated in Figure 1, this mismatch is explained by the phase variation *φ_xy_* along the slice profile, causing either a faster dephasing or a short period of rephasing followed by dephasing. Consequently, depending on the pulse shape and effective flip angle, the polarity of the gradient *G_slice_* must be included for modeling, as switching polarity inverts *φ_xy_* and thus signal dephasing.

In addition to the polarity dependency of *G_slice_*, the effects of $B_{1}^{+}$ variations and scaling of the slice profile with λ have been investigated in model *S*_4_. However, changes in $R_{2}^{*}$ due to $B_{1}^{+}$ and λ were relatively small compared with *S*_3_ (Table 1). Evaluation has been performed under the assumption that with an ideal model the estimated $R_{2}^{*}$ maps should be independent of *G_slice_* polarity. For the models *S*_1_ and *S*_2_, strong differences between *G_slice_* polarities were found, primarily due to *φ_xy_*, and by using *S*_3_ it was substantially reduced, which indicates improved modeling. However, the main challenge for validation of the models was that, in vivo, no ground truth was available.

Another important aspect is the assumption that TR for a given *α* is sufficiently long to avoid T_1_ influence in the presence of *G_z_*. The experimental results in Figure 5A are in accordance with the simulation results in Figure 2D, where the error decreases with TR/T_1_, and the minimum TR/T_1_ required enlarges with *α*. To gain SNR, it is desirable to use *α*_Ernst_, but care should be taken to prevent potential errors due to T_1_ and $B_{1}^{+}$. By increasing TR/T_1_, both the *α_Ernst_* and the overall SNR increase; however, the errors due to $B_{1}^{+}$ are magnified. For example, as illustrated in Figure 2, when TR/T_1_ = 2, the error when neglecting T_1_ is about 1.2% for *α* = *α_Ernst_* = 77°. By comparing errors caused by $B_{1}^{+}$ variation, a deviation of ξ = 1.15 leads to errors in a similar range. Thus, without knowing T_1_, it is not possible to separate these effects, but it can be adjusted by the RF pulse shape. For instance, to estimate $R_{2}^{*}$ more accurately, longer RF pulses can be used to obtain a slice profile closer to the ideal, rectangular shape. This would have the advantage that signal dephasing is influenced less by $B_{1}^{+}$ and TR/T_1_, but it would lead to stronger *φ_xy_* variations and zero crossings due to the sinc‐shaped signal decay in the presence of *G_z_*. However, for MWF estimation, very short pulses are needed, which will be more sensitive to these factors. Optimization of the RF pulses for specific applications was beyond of the scope of this work, but different pulses and their effects can be included and studied with the provided framework.

When comparing different modeling approaches, we can distinguish between models that fit parameters of F(*t*) from the signal decay^19^, ^21^ and models that use information from the pulse and field map to calculate F(*t*).^22^, ^23^, ^24^ Approaches that fit F(*t*) are more flexible in terms of model deviations from the ideal slice profile. For example, the sinc function used in the model approach by Fernandez‐Seara and Wehrli^19^ is well‐suited to model a variety of signal decays observed with different excitation pulses. Similarly, when modeling the macroscopic field as a quadratic function, the effects of a nonideal slice profile are inherently compensated for.^21^ However, in these models, the parameter estimation is often challenging due to the multiplication of F(*t*) with *S*_tissue_(*t*), thereby requiring the acquisition of many echoes. In contrast, with the analytical solution or our proposed numerical approach for F(*t*), only the parameters of the tissue model *S*_tissue_ need to be estimated. Thus, if the properties of the RF pulse are available, a detailed description of F(*t*) is possible, favoring a closed or numerical solution. To select an appropriate model for a certain RF pulse and flip angle, the provided framework can be used to evaluate the expected error of different modeling approaches. If *φ_xy_* might be neglected for a specific RF pulse and flip angle, then an analytic solution yields a faster solution of F(*t*).

This work has similar limitations as other related postprocessing approaches.^19^, ^20^, ^22^, ^24^, ^26^, ^29^ The assumption of a linear varying magnetic field in slice direction might not hold in some areas with large susceptibility changes, which is especially pronounced at higher field strengths. However, as we have solved the dephasing along the slice direction by numerical integration, the model can also easily be adapted to describe the dephasing also for a quadratic varying magnetic field. Furthermore, in‐plane dephasing effects are neglected. In 2D acquisitions the slice thickness is usually much larger than the in‐plane resolution, but this might reduce accuracy in areas where the macroscopic in‐plane field variations are high. A possible solution to account for in‐plane dephasing could be to calculate the voxel spread function in‐plane as proposed by Yablonskiy et al^51^ and multiply the result with F_3_ or F_4_, respectively. Given that *G_z_* is rather strong and that the signal dephasing is driven primarily by *G_z_*, a reliable parameter estimation is difficult to achieve due to the fast signal decay. To overcome this issue, for MWF and $R_{2}^{*}$ it has been shown that z‐shim gradients between echoes can improve maps by rephasing the signal with appropriated compensation gradients.^27^, ^52^ Therefore, future work will focus on extending our model by including the moment of the z‐shim gradients in the modeling to describe the signal dephasing accordingly for every echo.

In addition to variations of the macroscopic field, variation of the phase offset *φ*_0_ at TE = 0 could potentially influence signal dephasing. Contributions to *φ*_0_ in phased array coils can be divided into receive coil–dependent (receive sensitivity $B_{1}^{-}$) and receive coil–independent (e.g., $B_{1}^{+}$ phase).^53^ To reconstruct the navigator‐corrected raw data, a multi‐echo approach was used to combine the individual coil data.^38^ In this approach, for each coil, images from all echoes are multiplied with the complex conjugate of the first echo, which removes inherently all components of *φ*_0_ of the coil combined data. The development of the proposed models pointed out that the use of navigator echoes is highly recommended to compensate for phase errors arising from physiological fluctuations. As illustrated in Supporting Information Figure S2, depending on the subject’s reconstruction of parameter maps, not having the navigator echoes caused similar artifacts, as reported by Nam et al.^54^ If variations of *φ*_0_ should be included, a ROEMER/SENSE reconstruction could be applied, as an example.^55^, ^56^

The scan time of the proposed applications is about 6 minutes for $R_{2}^{*}$ maps and 12 minutes for MWF maps. This is clinically acceptable for whole‐brain investigations, but further investigations will also focus on combination with accelerated imaging methods such as 2D CAIPIRINHA.^57^

## 5 CONCLUSIONS

Proper modeling of the signal dephasing in the presence of *G_z_* for larger flip angles requires the consideration of |*M_xy_*| and *φ_xy_* with correct *G_slice_* polarity. Furthermore, $B_{1}^{+}$ and λ variations can potentially lead to a bias in the estimated model parameters, depending on the excitation pulse. Consequently, the proposed model allows to minimize the effects of *G_z_*, which is highly relevant for accurate $R_{2}^{*}$ and MWF mapping of the entire brain based on 2D mGRE.

## Supporting information


**FIGURE S1** Coronal $R_{2}^{*}$ maps from the phantom measurements (*α* = 90°) estimated for a varying slice‐selection gradient *G_slice_* within the model. The most homogenous map was obtained with *G_slice_* = 8.5 mT/m
**FIGURE S2** A,B, The MWF maps from 2 subjects. Maps are shown without and with correction of the raw data with the phase of the navigator echo
**TABLE S1** Influence of pulse shape and flip angle for modeling $R_{2}^{*}$. Note: The $R_{2}^{*}$ values (s^-1^) were estimated with models *S*_1_ to *S*_4_ from mGRE data acquired with 4 different pulses and *α* = 30° and *α* = 85°. It shows a flip angle and pulse shape dependency for *S*_1_ in all regions. By applying *S*_2_, differences decrease but $R_{2}^{*}$ values remain larger for *α* = 85° than for *α* = 30°. With *S*_3_ and *S*_4_, the flip angle dependency can be improved, leading to minimal differences of $R_{2}^{*}$ between the pulses. In the *S*_4_ model, $B_{1}^{+}$ and λ have a small additional effect on $R_{2}^{*}$ estimation, compared with *S*_3_
**TABLE S2** Myelin water fraction values (%) with models *S*_1_, *S*_3_, and *S*_4_ in different white matter regions for 10 subjects. Note: The MWF values are shown as median (interquartile range). The corresponding |*G_z_*| values are listed as mean (SD)
