# Supplementary material for: Assessment and correction of macroscopic field variations in 2D spoiled gradient‐echo sequences
Source: Magn Reson Med. 2019 Dec 23;84(2):620–33. doi: 10.1002/mrm.28139 (PMC7216950; doi:10.1002/mrm.28139)
Supplement: Supplementary file 1 — FIGURE S1 Coronal R2∗ maps from the phantom measurements (α = 90°) estimated for a varying slice‐selection gradient Gslice within the model. The most homogenous map was obtained with Gslice = 8.5 mT/m FIGURE S2 A,B, The MWF maps from 2 subjects. Maps are shown without and with correction of the raw data with the phase of the navigator echo TABLE S1 Influence of pulse shape and flip angle for modeling R2∗. Note: The R2∗ values (s-1) were estimated with models S 1 to S 4 from mGRE data acquired with 4 different pulses and α = 30° and α = 85°. It shows a flip angle and pulse shape dependency for S 1 in all regions. By applying S 2, differences decrease but R2∗ values remain larger for α = 85° than for α = 30°. With S 3 and S 4, the flip angle dependency can be improved, leading to minimal differences of R2∗ between the pulses. In the S 4 model, B1+ and λ have a small additional effect on R2∗ estimation, compared with S 3 TABLE S2 Myelin water fraction values (%) with models S 1, S 3, and S 4 in different white matter regions for 10 subjects. Note: The MWF values are shown as median (interquartile range). The corresponding |Gz| values are listed as mean (SD) [file MRM-84-620-s001.docx]

**Supporting information:**


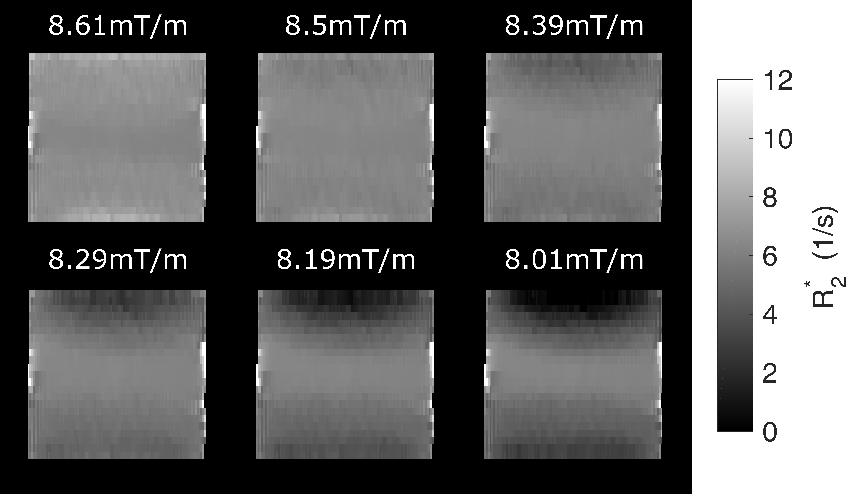


Supporting Information Figure S1: Coronal R_2_^*^-maps from the phantom measurements (α = 90°) estimated for a varying slice selection gradient G_slice_ within the model. The most homogenous map was obtained with G_slice_ = 8.5 mT/m.


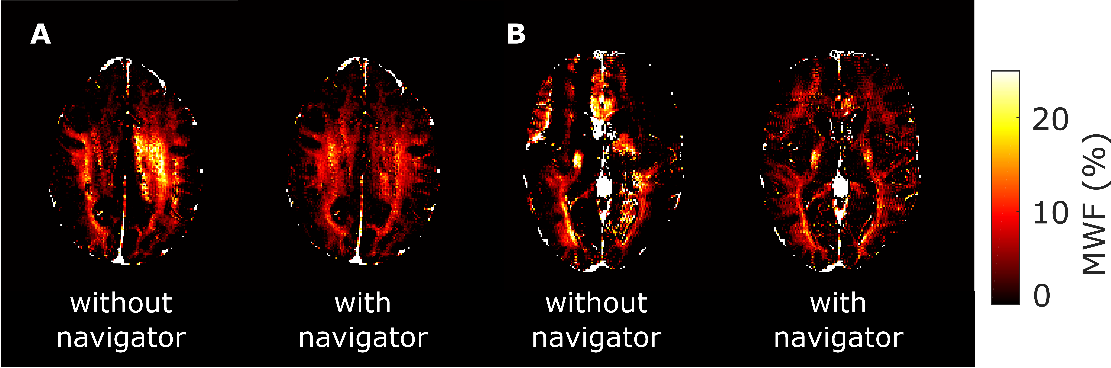


Supporting Information Figure S2: MWF-maps from two subjects (A) and (B). Maps are shown without and with correction of the raw data with the phase of the navigator echo.

Supporting Information Table S1: Influence of pulse shape and flip angle for modelling R_2_^*^. R_2_^*^ values (1/s) were estimated with models S_1_ to S_4_ from mGRE data acquired with four different pulses and α = [30°, 85°]. It shows a flip angle and pulse shape dependency for S_1_ in all regions. By applying S_2_, differences decrease but R2* values remain larger for α = 85° than for α = 30°. With S_3_ and S_4_ the flip angle dependency can be improved leading to minimal differences of R_2_^*^ between the pulses. In S_4_, B_1_^+^ and λ have a small additional effect on R_2_^*^ estimation, compared to S_3_.

|  |  | Global WM | | | | | | | |  | Caudate Nucelus | | | | | | | |
| --- | --- | --- | --- | --- | --- | --- | --- | --- | --- | --- | --- | --- | --- | --- | --- | --- | --- | --- |
| Pulse | α | S_1_ | | S_2_ | | S_3_ | | S_4_ | |  | S_1_ | | S_2_ | | S_3_ | | S_4_ | |
| Gauss | 30° | 26.62 | (9.00) | 19.94 | (4.26) | 19.85 | (4.29) | 19.85 | (4.28) |  | 25.32 | (3.76) | 23.13 | (3.38) | 23.15 | (3.36) | 23.14 | (3.37) |
|  | 85° | 28.41 | (10.07) | 21.46 | (4.44) | 19.80 | (4.03) | 19.27 | (4.26) |  | 25.70 | (3.95) | 23.42 | (3.58) | 23.49 | (3.18) | 23.41 | (3.15) |
| sinc-Hanning  BWT=2 | 30° | 25.46 | (8.25) | 19.50 | (4.07) | 19.71 | (4.07) | 19.69 | (4.07) |  | 24.87 | (3.32) | 22.95 | (3.18) | 23.07 | (3.14) | 23.07 | (3.14) |
|  | 85° | 27.33 | (9.46) | 21.12 | (4.64) | 20.07 | (4.18) | 19.59 | (4.28) |  | 25.24 | (3.90) | 23.18 | (3.67) | 23.42 | (3.30) | 23.36 | (3.27) |
| sinc-Hanning BWT=2.7 | 30° | 25.28 | (8.55) | 19.47 | (4.03) | 19.61 | (4.03) | 19.61 | (4.03) |  | 24.75 | (3.41) | 22.89 | (3.32) | 23.00 | (3.28) | 23.00 | (3.28) |
|  | 85° | 26.94 | (8.74) | 20.84 | (4.19) | 19.71 | (3.90) | 19.26 | (4.03) |  | 25.30 | (3.72) | 23.37 | (3.53) | 23.53 | (3.17) | 23.46 | (3.15) |
| sinc-Hanning  BWT=8 | 30° | 23.40 | (6.64) | 19.46 | (3.84) | 19.43 | (3.85) | 19.41 | (3.84) |  | 24.14 | (2.94) | 22.98 | (2.89) | 23.00 | (2.88) | 22.99 | (2.88) |
|  | 85° | 24.89 | (7.06) | 20.83 | (4.02) | 19.81 | (3.76) | 19.49 | (3.83) |  | 24.38 | (3.31) | 23.12 | (3.42) | 23.30 | (3.04) | 23.28 | (3.03) |
|  |  |  |  |  |  |  |  |  |  |  |  |  |  |  |  |  |  |  |
| Mean (standard dev.) |  | 26.04 | (1.59) | 20.33 | (0.82) | 19.75 | (0.19) | 19.52 | (0.20) |  | 24.96 | (0.52) | 23.13 | (0.19) | 23.24 | (0.22) | 23.21 | (0.19) |
|  |  |  |  |  |  |  |  |  |  |  |  |  |  |  |  |  |  |  |
|  |  | Putamen | | | | | | | |  | Thalamus | | | | | | | |
| Pulse | α | S_1_ | | S_2_ | | S_3_ | | S_4_ | |  | S_1_ | | S_2_ | | S_3_ | | S_4_ | |
| Gauss | 30° | 30.90 | (4.84) | 25.27 | (4.22) | 25.19 | (4.22) | 25.16 | (4.22) |  | 26.95 | (3.85) | 19.70 | (4.37) | 19.58 | (4.40) | 19.50 | (4.41) |
|  | 85° | 32.49 | (5.51) | 26.98 | (4.24) | 25.44 | (3.97) | 24.74 | (3.91) |  | 29.35 | (4.47) | 22.31 | (4.15) | 20.22 | (4.33) | 18.63 | (4.60) |
| sinc-Hanning  BWT=2 | 30° | 29.98 | (4.56) | 24.82 | (4.08) | 25.05 | (4.07) | 25.01 | (4.07) |  | 25.64 | (3.61) | 19.03 | (4.19) | 19.29 | (4.18) | 19.19 | (4.19) |
|  | 85° | 31.85 | (5.01) | 26.62 | (4.06) | 25.59 | (4.03) | 24.94 | (4.03) |  | 28.06 | (4.16) | 21.42 | (4.05) | 20.00 | (4.19) | 18.57 | (4.42) |
| sinc- Hanning BWT=2.7 | 30° | 29.71 | (4.39) | 24.69 | (4.04) | 24.84 | (4.03) | 24.81 | (4.02) |  | 25.50 | (3.63) | 19.08 | (4.20) | 19.24 | (4.20) | 19.17 | (4.20) |
|  | 85° | 31.39 | (5.10) | 26.34 | (4.10) | 25.23 | (3.89) | 24.61 | (3.82) |  | 28.03 | (4.19) | 21.54 | (4.16) | 20.02 | (4.31) | 18.62 | (4.53) |
| sinc-Hanning  BWT=8 | 30° | 27.91 | (4.02) | 24.55 | (3.88) | 24.50 | (3.87) | 24.46 | (3.87) |  | 23.17 | (3.43) | 18.89 | (3.96) | 18.83 | (3.96) | 18.75 | (3.96) |
|  | 85° | 29.49 | (4.56) | 26.21 | (4.12) | 25.14 | (3.90) | 24.66 | (3.84) |  | 25.51 | (3.90) | 21.35 | (4.13) | 19.94 | (4.23) | 18.91 | (4.36) |
|  |  |  |  |  |  |  |  |  |  |  |  |  |  |  |  |  |  |  |
| Mean (standard dev.) |  | 30.46 | (1.48) | 25.69 | (0.96) | 25.12 | (0.34) | 24.80 | (0.23) |  | 26.53 | (1.96) | 20.41 | (1.38) | 19.64 | (0.49) | 18.92 | (0.34) |

Supporting Information Table S2: MWF values (%) with models S_1_, S_3_ and S_4_ in different WM regions for 10 subjects. MWF values are shown as median (interquartile range). In addition, corresponding |Gz| values are listed as mean (standard deviation).

| Region | S_1_ | | S_3_ | | S_4_ | | G_z_ (µT/m) | |
| --- | --- | --- | --- | --- | --- | --- | --- | --- |
| Genu corpus callosum | 4.37 | (3.93) | 12.09 | (5.91) | 12.66 | (5.98) | 54.53 | (10.84) |
| Body corpus callosum | 3.70 | (3.15) | 6.65 | (1.90) | 6.67 | (2.00) | 9.81 | (3.41) |
| Splenium corpus callosum | 14.10 | (2.32) | 14.23 | (2.24) | 14.03 | (2.06) | 4.93 | (1.41) |
| Superior corona radiata | 7.06 | (2.04) | 8.20 | (1.72) | 8.22 | (1.69) | 5.28 | (1.96) |
| Posterior corona radiata | 7.14 | (1.41) | 7.34 | (1.55) | 7.34 | (1.53) | 3.28 | (1.25) |
| Superior longitudinal fasciculus | 8.71 | (1.19) | 8.94 | (0.91) | 8.93 | (0.92) | 4.58 | (1.10) |
